# Porous Thermal Insulation Polyurethane Foam Materials

**DOI:** 10.3390/polym15183818

**Published:** 2023-09-19

**Authors:** Zhiguo Wang, Chengzhu Wang, Yuebin Gao, Zhao Li, Yu Shang, Haifu Li

**Affiliations:** 1 College of New Energy, Xi’an Shiyou University, Xi’an 710065, China; zhgwang@xsyu.edu.cn (Z.W.); wangchengzhu0516@163.com (C.W.); 180303@xsyu.edu.cn (Y.S.); 2 Research Institute of Petroleum Exploration & Development, PetroChina, Beijing 100083, China; gaoyb69@petrochina.com.cn; 3 Shaanxi Haichuang Industrial Co., Ltd., Xi’an 712034, China; haichuanglhf@163.com

**Keywords:** porous thermal insulation materials, polyurethane foam, structures and properties, preparation methods, performance characterization

## Abstract

Porous thermal insulation materials (PTIMs) are a class of materials characterized by low thermal conductivity, low bulk density and high porosity. The low thermal conductivity of the gas enclosed in their pores allows them to achieve efficient thermal insulation, and are they among the most widely used and effective materials in thermal insulation material systems. Among the PTIMs, polyurethane foam (PUF) stands out as particularly promising. Its appeal comes from its multiple beneficial features, such as low density, low thermal conductivity and superior mechanical properties. Such attributes have propelled its broad application across domains encompassing construction, heterogeneous chemical equipment, water conservation and hydropower, and the aviation and aerospace fields. First, this article outlines the structure and properties of porous thermal insulation PUF materials. Next, it explores the methods of preparing porous thermal insulation PUF materials, evaluating the associated advantages and disadvantages of each technique. Following this, the mechanical properties, thermal conductivity, thermal stability, and flame-retardant characteristics of porous thermal insulation PUF materials are characterized. Lastly, the article provides insight into the prospective development trends pertaining to porous thermal insulation PUF materials.

## 1. Introduction

Porous thermal insulation materials (PTIMs) are energy-saving and consumption-reducing composite materials that are widely used for thermal insulation and heat preservation in fields such as construction, shaped chemical equipment, aerospace, water conservation and hydropower [1]. They are ideal thermal insulation materials with the following characteristics: (1) good flame-retardant properties with high safety, excellent fire resistance, and a long product lifecycle [2]; (2) low thermal conductivity [2]; and (3) a short production cycle, good water resistance, and crack resistance. These advantages of porous insulation materials can effectively save costs and improve the efficiency of engineering operations [3].

Polyurethane (PU) is a high–molecular–weight polymer prepared by combining multiple organic cyanates and polyol compounds with other additives. With the advantages of having a low density and being lightweight, as well as good thermal insulation, low thermal conductivity, and a large thermal storage coefficient, PU materials are widely used in engineering fields for thermal insulation and heat preservation. Its versatility and multifunctionality are important features that distinguish PU from other polymer materials. PU porous materials (PUPMs) are the most widely used, accounting for more than 50% of the total production of PU materials. Due to advances in science and technology, PU porous foam materials are being increasingly used for thermal insulation in fields such as biomedicine, construction engineering, aviation, aerospace, among others [4].

The porosity of PU porous foam materials can exceed 95%, which is mainly due to its closed pores. Compared with expanded polystyrene (EPS) foam [5], the heat insulation and strength of PU porous foam exceeds that of EPS foam. Its compressive strength is 0.196 MPa and its thermal conductivity is 0.0233–0.0256 W/(m·K). The applicable temperature range is wider (−110–200 °C), with lower water absorption (about 0.2%), better anti–corrosion properties, and strong bonding performance. Its weather resistance and dimensional stability are better than those of polystyrene foam. However, its flammable nature makes it extremely limited in engineering applications. Therefore, researchers hope to improve the flame-retardant properties of PU insulation by adding functional flame retardants [6].

Based on this requirement, this study reviews the structure and properties of porous thermal insulation PU foams (PUFs) from the perspective of the application of PTIMs. We focused on the methods of preparing porous thermal insulation PUFs and compared the advantages and disadvantages of the preparation methods. In addition, the mechanical properties, thermal conductivity, thermal stability, and flame retardancy of porous thermal insulation PUF materials were characterized. Finally, progress regarding research into porous heat insulation materials made from PUF is summarized, and the direction of future developments is discussed.

## 2. Structure and Properties of the Porous Thermal Insulation PUF Materials

### 2.1. Structure of the PUF Materials

**Figure 1:**
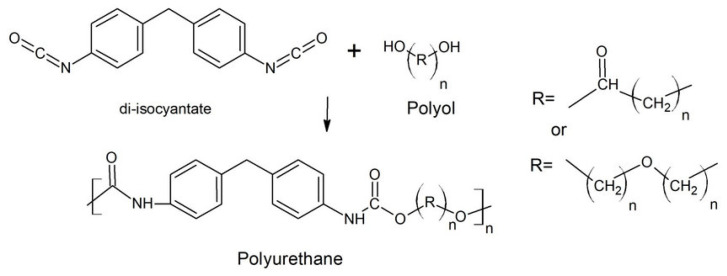
Schematic of the reaction for the synthesis of PU.

**Figure 2:**
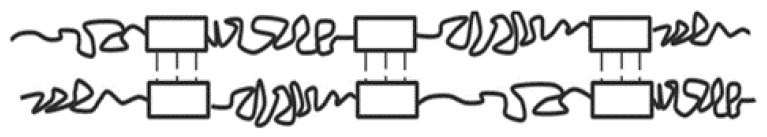
Representation of the soft and hard segments [8].

PU is a polymer composed of organic units linked by urethane, which is usually produced by reacting isocyanates with polyols (see Figure 1). Polyols and isocyanates form different structural domains in PU, which are divided into hard and soft chain segments. These chain segments determine the properties of the PU material, such as its softness, flexibility or hardness [7]; the soft and hard segments are shown in Figure 2. In the structure of PU, polyols create long and flexible chain segments, called soft chain segments, which provide the polymer with softness and high elasticity. The long chains and low crosslinking of polyols make the polymer highly elastic, generating soft PU materials. Modulating the degree of the polyols’ long-chain crosslinking allows the polymer to be used in the manufacturing of PUF materials [8]. PUFs are generally classified as rigid PUFs (RPUFs) and flexible PUFs (FPUFs). The flexibility or rigidity of a PUF is primarily determined by the type and amount of chemicals used during the synthesis of the foam. FPUFs are typically made with polyols that have a relatively low molecular weight and are often referred to as low molecular weight polyols. These polyols are more flexible and have greater mobility, allowing the foam to bend and compress easily. RPUFs usually use diisocyanates that have a higher molecular weight and are referred to as high-functionality diisocyanates. The higher functionality means that more crosslinking can occur during the reaction, resulting in higher rigidity and a more structured foam. The ratio of polyols to diisocyanates and the specific formulation used (including the use of catalysts, blowing agents and additives) also play significant roles in determining the properties of the foam. In a flexible foam, the polyol to diisocyanate ratio is usually higher, allowing for more flexibility in the foam’s structure. By contrast, rigid foam formulations have a higher diisocyanate content, leading to a more rigid and stable structure. Blowing agents are used to create the cellular structure in PUFs. Flexible foams typically use physical blowing agents, such as water, which generate smaller cells and more open structures, contributing to their flexibility. In a rigid foam, chemical blowing agents or physical blowing agents with higher boiling points are used to create larger cells and a structure with more closed cells, resulting in a more rigid foam. The degree of crosslinking between the polyurethane polymer chains and the overall density of the foam also affect the flexibility and rigidity. Greater crosslinking and a higher density create a more rigid foam, whereas less crosslinking and a lower density create a more flexible foam. 

### 2.2. Properties of PUF Materials

#### 2.2.1. Physical Properties

PUFs have good mechanical properties, such as high tensile strength, tearing resistance, and abrasion resistance, which increases with the hardness of the PUF [9]. In addition, PUFs have excellent resistance to mineral oil, grease, gasoline, organic solvents, and acidic and alkaline solutions [9]. When PUFs made from aromatic isocyanates are exposed to UV light, yellowing occurs because they contain chromophores that interact with light, and the degree of yellowing depends on the intensity of UV radiation, but the yellowing barely affects their physical properties [10]. In addition, non-yellowing foams can be manufactured by using aliphatic isocyanates, but they are more expensive than regular foams. The physical properties of PUF materials are highly tunable, and by controlling the ratio between the hard crystalline and soft non-crystalline segments, PU materials can be made with different physical properties, and their overall physical properties, such as resistance to wear, temperature resistance, sealing, sound insulation, processing properties, and degradability, are excellent.

#### 2.2.2. Chemical Properties

PU materials are mostly chemically inert and they are usually non-toxic. However, these compounds are classified as combustible substances and must be kept away from open flames. Moreover, the combustion of PU produces large amounts of carbon monoxide (CO), nitrogen oxides (NO_x_), hydrogen cyanide (HCN), and other asphyxiating gases, which greatly increase the toxicity of the fumes of combustion; this poses a fatal threat to fire victims [11], so PUFs usually require treatment with a flame retardant during their preparation.

## 3. Preparation of Porous Thermal Insulation PUF Materials

### 3.1. Traditional Foaming Technologies

#### 3.1.1. Solution Casting/Salt Precipitation Method

**Figure 3:**
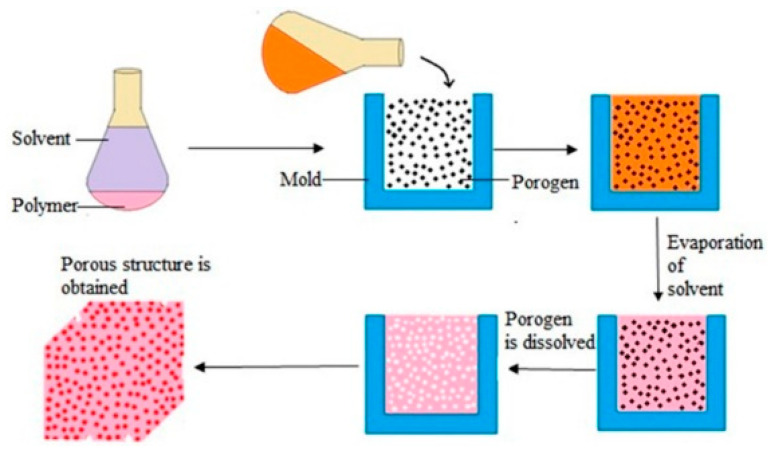
Schematic of the solvent casting and particle leaching process [21].

The solution casting/salt precipitation method [12] (see Figure 3) is a simple process used for the preparation of porous materials and it does not require special equipment. The method is as follows. First, a polymer solution with a mass fraction of 5–20% is prepared. Secondly, particles with a specific diameter are added to the polymer solution, and then, the polymer containing the particles is cured by air-drying, vacuum-drying, or freeze-drying to obtain the polymer foam. Finally, the polymer foam is immersed in water or other solvents to leach out the particles. The porous structure of the PUPMs prepared by solution casting/salting depends on the shape, size, number of leached particles, and the initial concentration of the polymer solution.

Asadpour et al. [13] obtained composite scaffolds of porous polyurethane and polycaprolactone (PCL) by utilizing the solution casting/salt precipitation method; the final composite scaffolds of polyurethane had good porosity (94 ± 1%) and the pores’ size ranged from 142 μm to 170 μm.

#### 3.1.2. Phase Separation Method

The phase separation method is divided into two main categories, namely, thermally induced phase separation (TIPS) and non-solvent-induced phase separation (NIPS) [14]. TIPS achieves phase separation by lowering the temperature, whereas NIPS achieves phase separation by adding a non-solvent. TIPS takes advantage of the difference in the solubility of polymers at different temperatures to produce a homogeneous solution at high temperatures, then, the system is separated by lowering the temperature and adding a volatile extractant to extract the solvent. The extractant is removed by evaporation or freeze-drying to obtain the porous structure. The process consists of quenching the PU polymer solution below the solvent’s freezing point (T_k_) and inducing the separation of the liquids to form two phases, as follows: a polymer-rich phase and a polymer-deficient phase. The polymer-rich phase solidifies, whereas the polymer-deficient phase crystallizes. The removal of the crystalline fraction results in a highly porous structure (>90%) [15].

The structure of PUPMs depends on the concentration of the polymer solution, the quenching temperature, and the cooling rate. The higher the concentration of the polymer solution, the smaller the porosity and the size of the pores of the final material [16]. On the one hand, with a higher concentration, the less solvent there will be in the system, fewer crystals will be formed, and the porosity will be lower. On the other hand, because the increase in concentration increases the viscosity of the system, this increases the shear force on the crystals and it prevents the formation of large crystals, thus forming smaller crystals, and eventually, a smaller microporous structure for the material. At lower temperatures, solute nucleation is faster, but the growth of crystals is limited, eventually forming a large number of small crystals and small pore sizes, whereas at higher temperatures, the nucleation rate is limited and the growth of crystals increases, eventually forming larger crystals and larger pore sizes.

Guan et al. [17] analyzed the effect of the quenching temperature and the concentration of the polymer solution on the resulting pore size. The results showed that at the same polymer concentration, with a lower temperature, smaller pores were obtained. At temperatures of −20 °C, −80 °C, and −196 °C, the pore sizes of the materials were 36–203 μm, 23–154 μm, and 3–19 μm, respectively. At the same temperature, a higher polymer concentration led to a smaller pore size, with the rate of poring being 93.7%, 92.7%, and 85.9% and pore sizes of 89 μm, 62 μm, and 63 μm, at polymer mass fractions of 5%, 8%, and 10%, respectively.

Non-solvent phase separation methods are divided into three main categories, namely, solvent evaporation, vapor precipitation, and immersion precipitation [18]. In solvent evaporation, the polymer is dissolved into a solvent mixture consisting of a “good” solvent that is volatile and a “bad” solvent that is difficult to volatilize. The good solvent evaporates continuously, the polymer precipitates gradually, and the solution system undergoes phase separation and forms a microporous film. In the vapor precipitation method, the non-solvent vapor penetrates into the polymer solution and causes phase separation. The immersion precipitation method is more widely used, in which the polymer solution is scraped to a thin layer with a specific thickness on a supporting body and then immersed into a non-solvent, triggering the phase separation of the system via the exchange of the non-solvent and the solvent. Usually, the pore size of the porous PU obtained by non-solvent phase separation methods is only 1–2 μm, so a combination of salt precipitation and non–solvent phase separation is used to prepare PUPMs with pore sizes larger than 10 μm.

Gorna et al. [19] used dimethylformamide (DMF), dimethyl sulfoxide (DMSO), and N–methylpyrrolidone (NMP) as solvents; isopropanol, ethanol, and tetrahydrofuran (THF) were used as non–solvents; and sodium phosphate was used as a salting reagent to produce PUPMs with a porosity of up to 90% and an average pore size above 135 μm, with the DMF–THF system reaching an average pore size of 200 ± 45 μm.

#### 3.1.3. Melt Molding Method

Melt molding [20] is commonly used for the preparation of porous ceramics and metals, but it is also used for the preparation of polymeric materials, including PU. Porogenic agents and granular or powdered polymers are placed in a mold, then pressurized and heated above the transition temperature of glass (T_g_) to form a mold-shaped material. The material is then removed from the mold and the porogenic agent is leached out. The shape and size of the holes can be controlled, depending on the choice of different additives. From an industrial point of view, melt forming is the most convenient and economical way to prepare porous materials, as it allows for the rapid production of porous polymer materials of various shapes and sizes, but it is influenced by the complexity of the mold’s configuration and design. The advantages of melt forming are that it does not require the use of organic solvents to prepare the material, but the disadvantages are the presence of non-porous layers on the surface, the difficulty of leaching the porous formulations, and the need for high temperatures.

#### 3.1.4. Gas Foaming Method

**Figure 4:**
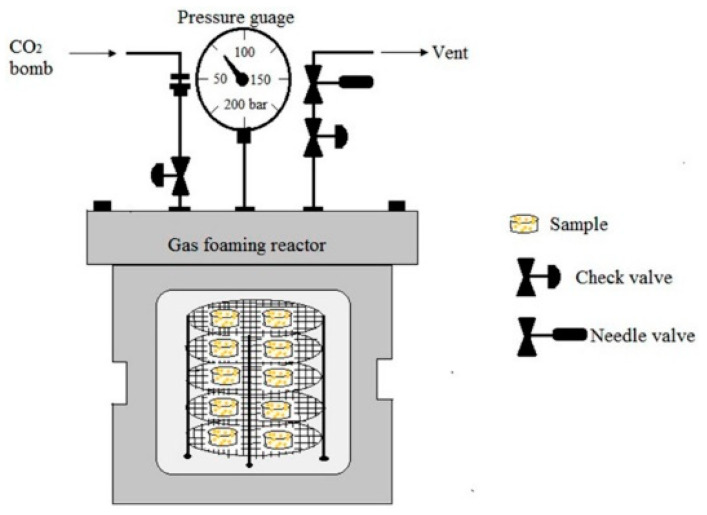
Schematic the CO_2_ gas foaming device [21].

Gas foaming [21] (see Figure 4) is a technique used to manufacture polymeric porous materials without the use of any organic solvent. Products manufactured using gas foaming are used in many fields such as aerospace, military industries, the automotive industry, railroad transportation, and the marine industry. The technology requires the use of high-pressure CO_2_ (800 psi) to fill the polymer with gas. When the polymer reaches gas saturation using high-pressure CO_2_, the intermolecular interactions between the CO_2_ and the polymer molecules increase, resulting in a lower glass transition temperature in the polymer. The rapid decompression leads to thermodynamic instability, as well as the formation of nucleated gas cells that form pores in the polymer matrix [21]. The advantage of gas foaming is that it does not require the use of organic solvents, and the disadvantage is that the size of the pores is difficult to control. Sun et al. [22] prepared thermoplastic PUFs using N_2_ and CO_2_ as co-blowing agents. It was shown that the density of the material could be reduced to 0.20 g/cm^3^ using N_2_ and CO_2_ as co-blowing agents.

#### 3.1.5. Freeze-Drying Method

**Figure 5:**
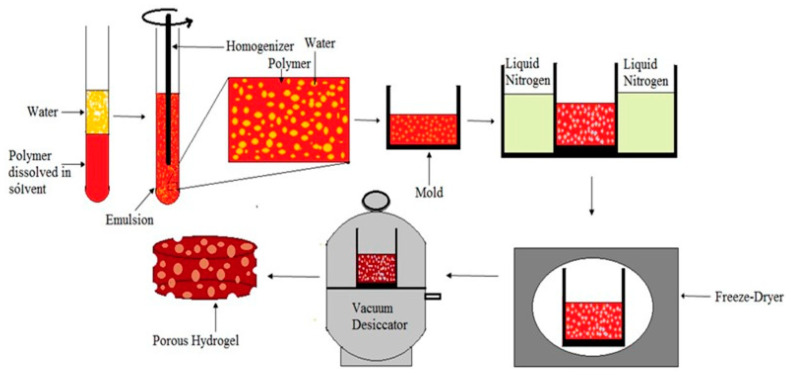
Schematic of the freeze-drying process [21].

The freeze-drying method (see Figure 5) is a common method used for the fabrication of porous materials. By freeze-drying, porous materials with a pore size of 20–200 μm and a porosity of more than 90% can be prepared [23]. This technique requires a homogeneous mixture of a solvent containing the polymer and water; this forms an emulsion with water in the dispersed phase, and it is followed by freeze-drying to maintain the liquid’s structure and to finally generate porous materials. The porosity and pore size are influenced by the concentration and viscosity of the polymer solution, the amount of the water phase dispersed in the system, and the cooling rate. As the concentration or viscosity of the continuous polymer increases, the sheer force that occurs during the dispersed aqueous phase increases, ultimately leading to a reduction in the porosity and pore size of the porous material. As the amount of water decreases, the average pore size also decreases. If the cooling rate is stable, the pores of the prepared material are more uniform. If the cooling rate is not uniform, the formation and growth of the water phase will be uneven, and the uneven transfer of heat will lead to changes in the structure of the porous material. The advantages of the freeze-drying method include the reduced use of toxic solvents and the shorter drying and leaching times of the porous components, whereas the disadvantages include the instability of the emulsion and the need for additional surfactants in the system [24].

#### 3.1.6. Casting Method

The preparation of PUFs using the casting method is an established technique for producing tailored foam structures [25]. Casting involves the intricate interplay of polyol and isocyanate, catalyzed by additives and propelled by a blowing agent. The resulting foam exhibits versatility, making it suitable for applications such as cushioning, insulation, and packaging. This technique offers the advantage of controlled mixing, enabling precise customization of the foam’s properties [26]. The casting method orchestrates the complex chemical reaction between polyol and isocyanate, catalyzed by additives, to yield PUF. The incorporation of a blowing agent initiates the formation of bubbles, resulting in the characteristic cellular structure. The kinetics and thermodynamics of this reaction demand careful consideration of parameters such as the polyol–isocyanate ratio, the concentration of the catalyst, and the curing temperature. Achieving optimal characteristics for the foam necessitates precise control over various parameters. Manipulating the polyol–isocyanate ratio enables regulation of the foam’s density and mechanical properties, whereas the choice of catalyst impacts the curing rate and the foam’s structure. The curing conditions, including the temperature and humidity, further shape the foam’s final properties. A comprehensive understanding of these factors is imperative for fine-tuning the foam’s attributes to meet the requirements of the specific application. The casting method’s advantages extend beyond controlled mixing and customization. Its adaptability to intricate shapes and the capacity to integrate additives for enhanced properties make it suitable for diverse applications. From providing cushioning in furniture and automotive components, to offering effective thermal insulation, PUFs generated via the casting method play a pivotal role in modern material engineering [27]. In conclusion, the casting method serves as a cornerstone for producing PUFs with tailored properties. A nuanced grasp of the interplay among the polyol, isocyanate, catalysts, and blowing agents is paramount for achieving the desired characteristics of the foam. The casting method’s versatility and adaptability underscore its relevance in meeting multifaceted industrial needs, further advancing the realm of materials in science and engineering.

#### 3.1.7. Spray-Foaming Method

PUFs prepared via the spray-foaming method offer exceptional versatility and performance across various industries. The spray-foaming technique [28] offers numerous advantages, including efficient mixing, rapid curing, and precise control over the foam’s density. The process involves the reaction of polyol and isocyanate, facilitated by a blowing agent, yielding a cellular structure with excellent thermal insulation properties. This technique offers distinct advantages over other methods due to its ability to produce a foam with a controlled density and enhanced thermal insulation properties. At the heart of the spray-foaming process lies the exothermic reaction between polyol and isocyanate. Blending these components while introducing a blowing agent results in the formation of a cellular structure. The blowing agent, typically a physical or chemical compound, vaporizes during the reaction, creating gas bubbles that expand the foam. The reaction’s kinetics and thermodynamics significantly impact the foam’s structure and properties; this requires precisely controlling parameters such as the temperature, mixing ratios, and curing time. Achieving optimal characteristics for the foam relies on controlling parameters such as the isocyanate-to-polyol ratio, the type and concentration of the blowing agent, and the curing conditions. Variations in these factors lead to differences in the cells’ size and the foam’s density, thermal conductivity, and mechanical properties. The careful manipulation of parameters enables us to tailor the properties of the foam for specific applications, enhancing its overall performance [29]. Recent advancements in spray-foaming technology include the development of eco-friendly blowing agents to address environmental concerns. Additionally, improved equipment designs allow for better control over the application of the foam, resulting in enhanced consistency and reduced waste. Researchers are also exploring ways to further improve the foam’s fire resistance and overall sustainability. Efficient mixing, rapid curing, and the adjustable properties of the foam make this method invaluable for applications ranging from construction to packaging. Understanding the interplay of parameters influencing the foam’s quality is essential for optimizing its performance. Ongoing research and technological advancements continue to enhance the spray-foaming process, ensuring its continued relevance and contribution to the science of modern materials.

#### 3.1.8. Reaction Injection Molding Method

The reaction injection molding (RIM) method has emerged as a prominent technique for manufacturing PUFs with tailored properties. RIM combines liquid polyol and isocyanate components, catalyzed by additives, within a mold to create foam structures [30]. The RIM process offers advantages such as rapid production, the ability to produce designs with intricate parts, and it provides the foam with consistent properties. RIM involves the dynamic interaction between the liquid polyol and isocyanate components, triggered by a catalyst, and propelled by injection into a mold. The ensuing exothermic reaction leads to the formation of bubbles, resulting in the characteristic cellular structure of PUFs. The RIM process’s unique attributes, such as the rapid cycle times and capacity for intricate mold designs, contribute to its effectiveness in producing foam components. The optimal characteristics of the foam are achieved through the careful control of the RIM process’s parameters. The choice of material, including the type of polyol and isocyanate, significantly impacts the foam’s properties. The molding temperature and the injection pressure govern the foam’s density, cell size, and mechanical attributes. Attention to these factors is essential to ensure consistent foam quality across production batches. The RIM method’s advantages extend beyond those of conventional foam production methods. Its ability to swiftly produce complex foam components with precise designs makes it suitable for industries ranging from the automotive field to electronics. The resulting foam exhibits exceptional mechanical strength, thermal insulation, and resistance to impact, underscoring its potential for widespread application [31]. In conclusion, the reaction injection molding method is a robust approach for preparing PUFs, enabling the production of tailored foam structures for diverse industrial applications. A comprehensive understanding of the interplay between the choice of materials, the processing parameters, and the design of the mold is crucial for achieving the desired properties of the foam. The RIM method’s efficiency, coupled with its ability to produce intricate foam components, underscores its significance in advancing materials for scientific and engineering applications.

### 3.2. Advanced Foaming Technologies

#### 3.2.1. Electrostatic Spinning Method

**Figure 6:**
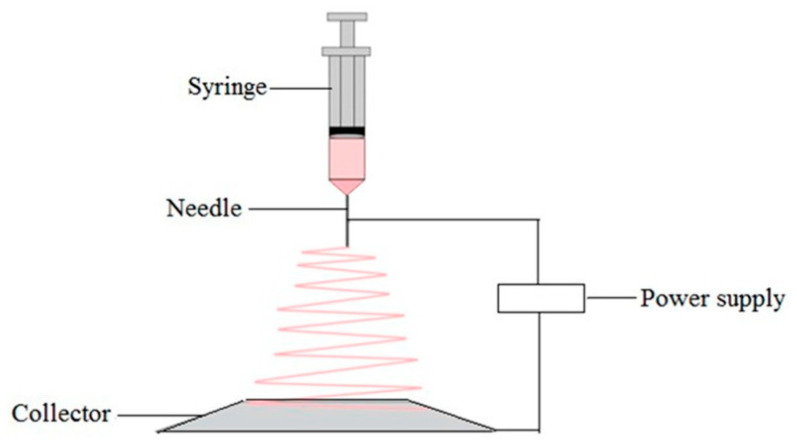
Schematic diagram of the electrostatic spinning technology [21].

The electrostatic spinning method (see Figure 6) is a new process used for the preparation of PUPMs, and it is unlike conventional methods. The principle is as follows: the polymer solution or melt is electrostatically charged with thousands to tens of thousands of volts, and the charged polymer droplets are accelerated through an electric field at the apex of the Taylor cone of the capillary. When the electric field’s force is large enough, the polymer droplets overcome the surface tension and form a jet stream that evaporates during the jet process, and eventually, it falls on the receiving device to form a fibrous porous material. In general, polymer fibers with diameters of several microns to 100 nm can be obtained via electrostatic spinning [32].

Andrews et al. [33] produced PU materials using polyether-based PU as the raw material by adjusting the parameters of electrostatic spinning, such as the polymer solution’s flow rate, the spinneret’s distance, and the voltage of the grid and spool. During the preparation of PU, their performance for specific applications was improved by adding graphene oxide (GO) and polylactic acid, etc. Xue et al. [34] prepared composite porous PU materials with different GO contents using electrostatic spinning, and they investigated the effect of the GO content on the properties of the porous materials. The results showed that the tensile strength, Young’s modulus, and hydrophilicity of the composites increased with the increase in the GO content. Kucinska-Lipka et al. [35] concluded that PUPMs are non-toxic and elastic, they have excellent biocompatibility, and their use in cellular tissues has great prospects for application. By choosing appropriate electrospinning parameters, suitable solvents and concentrations of PU solutions, fibers similar to natural soft tissues with suitable porosities, dimensions, and surface roughness can be prepared [36].

#### 3.2.2. Three-Dimensional Printing Technology

**Figure 7:**
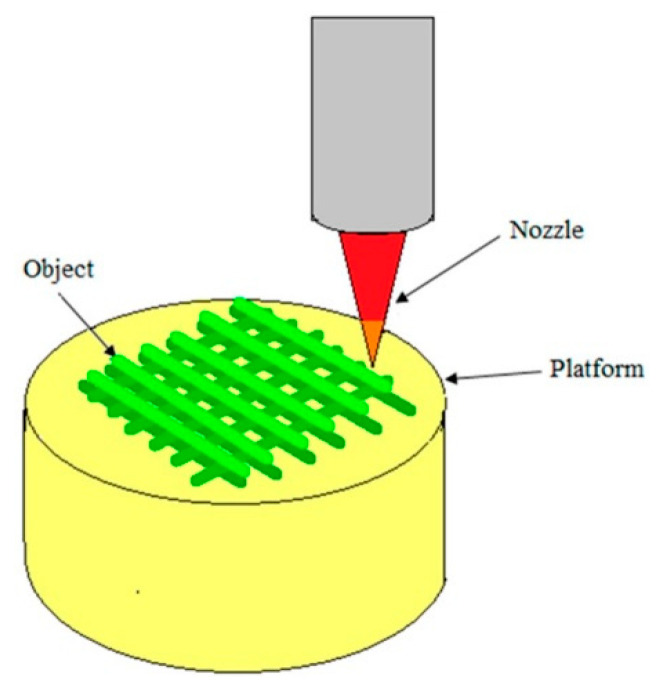
Schematic diagram of 3D printing technology [21].

Three-dimensional printing (3DP) technology [37] is a technique of formation that uses powdered, bondable materials (e.g., plastics, metals, etc.) to create solid objects based on digital models stacked layer by layer (see Figure 7). Fused deposition molding (FDM) is one of the most affordable technologies for implementing 3DP in terms of the printer’s purchasing and operating costs. Compared with other 3DP technologies, FDM also offers the possibility of using multiple materials in the printing process [37]. Three-dimensional printing technology shows great dynamism and potential due to its designability, reproducibility, repeatability, low cost, and high efficiency, which can be applied in various fields, such as biomedical, aerospace, automotive, and architectural engineering. Moreover, some progress has been made regarding the fabrication of PUPMs using 3DP technology.

Bates et al. [38] prepared a PUPM with a honeycomb structure using the 3DP technique, and they investigated the energy absorption of the honeycomb-structured PU material. It was shown that the material could absorb energy in the range of 0.01–0.34 J/cm³ under compression at strain rates of 0.03–0.3 s^−1^, and the maximum energy absorption of the material was 36%. This study shows that honeycomb-structured PU materials produced by 3DP have great potential for applications requiring energy absorption.

Sakkadech et al. [39] used 3DP technology to fabricate PU materials with an open-celled porous structure with 74% porosity. The researchers studied the mechanical and biological behavior of the internal structures of the 3D materials (octahedra, strut octahedra, cubes, and truncated octahedra) and found that the strut octahedra shape not only had greater stiffness and strength in terms of compression, shear, and torsion, but it also increased the rate of osteoblast proliferation. It was shown that bone implants could be fabricated using 3DP technology, and their mechanical and biological properties could be tailored by modifying the internal structure. Adriana et al. [40] prepared, for the first time, composite scaffolds grafted with poly(3–hydroxybutyrate) PU using 3DP technology, which could be used to improve the mechanical properties of biomimetic copolymers.

## 4. Characterization of the Porous Thermal Insulation PUF Materials

### 4.1. Characterization of the Mechanical Properties

**Figure 8:**
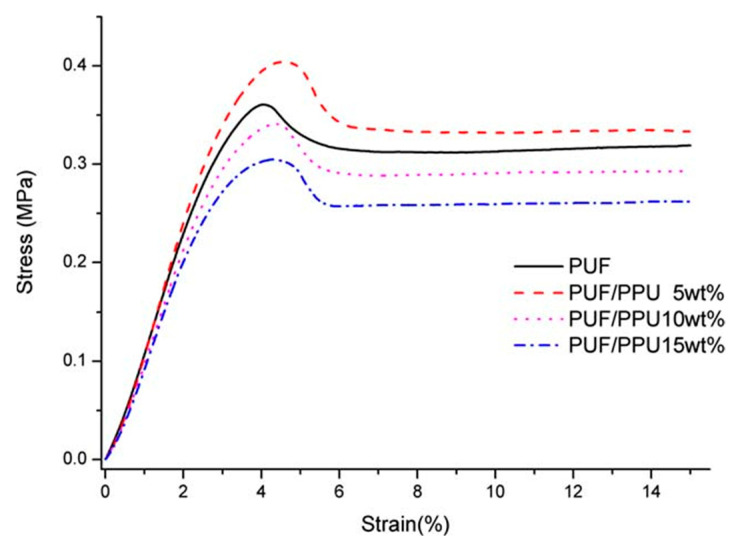
Compressive stress–strain curves of PUFs with different contents of PPU [48].

Due to the low flexural and tensile strength of RPUF, it cannot meet the requirements of most engineering projects. In order to improve its strength, scholars around the world have conducted in-depth research. Since PU is a polymer structure, scientific researchers have added additives without changing this polymer’s structure to improve the mechanical properties of PU. Currently, the main methods used to improve the mechanical properties are polymer alloys, fiber reinforcement, etc. Saha et al. [41] considered three different types of nanomaterials, namely, spherical TiO_2_, platelet nanoclay, and rod-like carbon nanofibers (CNFs), and they added the nanomaterials to PUF using the acoustic wave technique. Tensile tests were performed in accordance with ASTM D 638–89 [42], using a Type IV specimen with a thickness of 4 mm, a width of 12.7 mm, and a gage length of 64 mm. The length of the specimen lay along the in-plane direction of the foam, and the specimens were prepared using a sharp knife to form a dog-bone shape. Compression tests were performed on prismatic bar specimens with dimensions of 25.4 mm × 25.4 mm × 12.7 mm using a servo-hydraulic MTS testing system. Flexure tests were performed using the Zwick–Roell test machine with a 2.5 kN load cell at a crosshead speed of 2 mm/min. The tests were performed in accordance with ASTM D 790–86 [43]. The results showed that when only 1 wt% of CNFs was added to the PUF, the tensile modulus increased by about 86%, the compressive modulus increased by 40%, and the bending modulus increased by 45%, whereas the tensile strength increased by about 35%, the compressive strength increased by 57%, and the bending strength increased by 40%. The mechanical properties of the PUF were significantly enhanced. Yin et al. [44] added 2–6% of ZIF–8@Ti_3_C_2_T_x_ to FPUF. As a result, the tensile strength of the flame-retardant FPUF increased by 18.2%, 45.4%, and 52.7%. The mechanical performance of the flame-retardant FPUF was characterized using tensile and compression tests with a CMT6104 universal testing machine (Shenzhen Sand Material Testing Co. Ltd., Shenzhen, China). The tensile strength of the samples was characterized by the procedure of the ISO 1856 (2000) standard [45] at a 100 mm/min crosshead speed with dimensions of 120 × 25 × 10 mm^3^. The results showed that FPUF6 exhibited the best tensile strength, which was attributed to the large surface free energy of ZIF–8@Ti_3_C_2_T_x_ nanosheets [46] and the strong interfacial interaction with the FPUF polymer chains. Moreover, there are abundant functional groups on ZIF–8@Ti_3_C_2_T_x_ that can form hydrogen bonds [47]; therefore, ZIF–8@Ti_3_C_2_T_x_ can act as a rigid chain to effectively buffer stresses and produce an FPUF with better mechanical properties. Yang et al. [48] made RPUF and PUF scraps into a powder, which was used to enhance the mechanical properties of the RPUF and PUF. The compressive strength and modulus of the foams were determined using a universal testing machine (Sans CMT6104, Shenzhen, China), at room temperature, in accordance with the GB/T 8813–8818 test standard [49]. All measurements were performed perpendicularly, in the direction of the foam’s growth. The crosshead speed was set at 2 mm/min and the foams were pressed with 15% deflection. The compressive modulus of the PUF increased from 10.91 MPa for the original PUF to 12.40 MPa when 5% PPU was added. The compressive stress–strain curves of PUFs with different PPU contents are shown in Figure 8.

### 4.2. Characterization of the Thermal Conductivity

Nazeran et al. [50] synthesized PUF nanocomposites with efficient thermal insulation properties using RPUF as a matrix with different mass percentages of a SiO_2_ aerogel. The thermal conductivity of the silica aerogel and RPUF samples was measured by using a thermal conductivity meter (made in the electrical engineering faculty of Sahand University of Technology) and the needle probe method. With this technique, the needle probe was inserted into the sample, and then an energy pulse with a certain electrical power was applied for a specified period of time. By applying the pulse, the temperature of the probe was increased; by stopping it, its temperature decreased over time until it reached an ambient temperature. Then, the thermal conductivity of samples was determined from the temperature–time and thermal conductivity–time curves. The RPUF samples were cured at 50 °C for 24 h before the thermal conductivity tests were performed. The results showed that, compared with pure foam, the thermal conductivity of the composites made with four blowing agents, namely, CFC–11, cyclopentane, n–pentane, and n–hexane, (see Table 1), decreased with an increase in the content of SiO_2_ aerogel (from 0 wt% to 5 wt%) from 0.0209 W/(m·K) to 0.0171 W/(m·K), from 0.0314 W/(m·K) to 0.0268 W/(m·K), from 0.0301 W/(m·K) to 0.0257 W/(m·K), and from 0.0345 W/(m·K) to 0.0299 W/(m·K), respectively. Thus, the PUF nanocomposites that were synthesized via the addition of SiO_2_ aerogel had lower thermal conductivity and enhanced thermal insulation properties.

**Figure 9:**
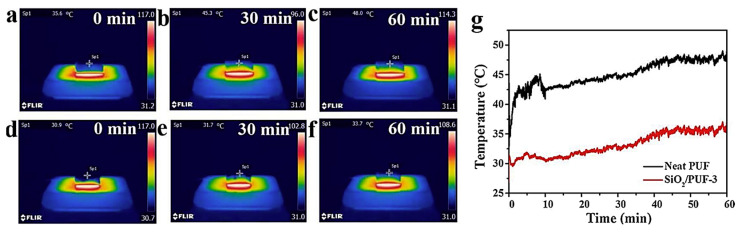
Thermal insulation properties of the pure PUF and SiO_2_/PUF–3, with a thickness of 2 cm during continuous heating in a 100 °C heater. (**a**–**c**) Color thermograms of the upper surface of clean PUF at different times from 0 min to 60 min. (**d**–**f**) Color thermograms of the upper surface of the SiO_2_/PUF–3 at different times from 0 min to 60 min. (**g**) Temperature profiles of each point on the upper surface over time [51].

Li et al. [51] used organic/inorganic hybrid and freeze-drying techniques to attach SiO_2_ aerogels to the surface of PU to prepare layered porous SiO_2_ PU composites; these techniques were able to largely retain the excellent properties of pure PU, such as thermal insulation and resistance to pressure, and they further improved the thermal insulation, compressive strength, and flame- and smoke-suppressing effects [52,53]. The thermal conductivity of the SiO_2_–PUF composites and pure PUF (30 × 30 × 10 mm^3^) was measured using a Hot Disk 2500–OT (Hot Disk, Sweden), in accordance with ISO 22007–2:2008 [54]. The PU composite effectively restricted air flow and heat conduction with its unique nanoporous structure [55], and the thermal conductivity decreased from 0.0309 W/(m·K) for pure PU to 0.0282 W/(m·K). Moreover, it gradually decreases with an increase in the content of the SiO_2_ aerogel (see Table 2). The color thermograms and temperature, versus the time curves of SiO_2_/PUF–3 and the pure PUF with a thickness of 2 cm during continuous heating at 100 °C in a heater, are recorded in Figure 9. It is noteworthy that the upper surface point temperature of SiO_2_/PUF–3 was always much lower than that of the pure PUF for the same heating time, indicating that the SiO_2_/PUF composite had superior thermal insulation properties. After heating at 100 °C for 60 min, the upper surface point temperature only increased from the initial 30.9 °C to 33.7 °C. This shows that adding silica aerogel to PUF is one of the more effective ways to improve the thermal insulation of PUFs.

**Figure 10:**
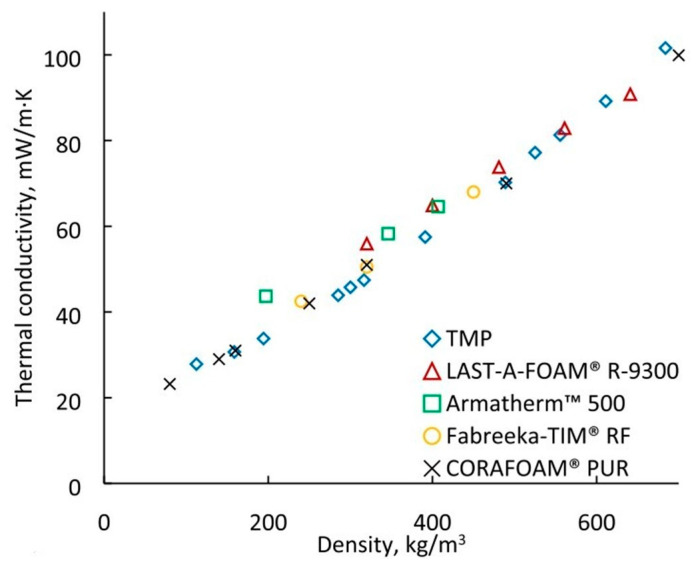
Thermal conductivity of TMP PUF compared with commercially available PUFs [56].

Andersons et al. [56] produced a rigid high-density PUF material using biopolyol derived from a renewable source of tall oil fatty acids. Disks with a diameter of 12.7 mm and a thickness of 2 mm were machined out of the monolithic trimethylolpropane PUF (TMP PUF) polymer and were used to determine its thermal conductivity, in accordance with the ASTM E–1461 standard [57], using an LFA 447 NanoFlash^®^Light Flash System. The polymer had a low thermal conductivity between 0.0205 W/(m·K) and 0.0264 W/(m·K), which is comparable to the thermal conductivity used for high-density insulating PUFs (see Figure 10).

### 4.3. Characterization of the Thermal Stability

**Figure 11:**
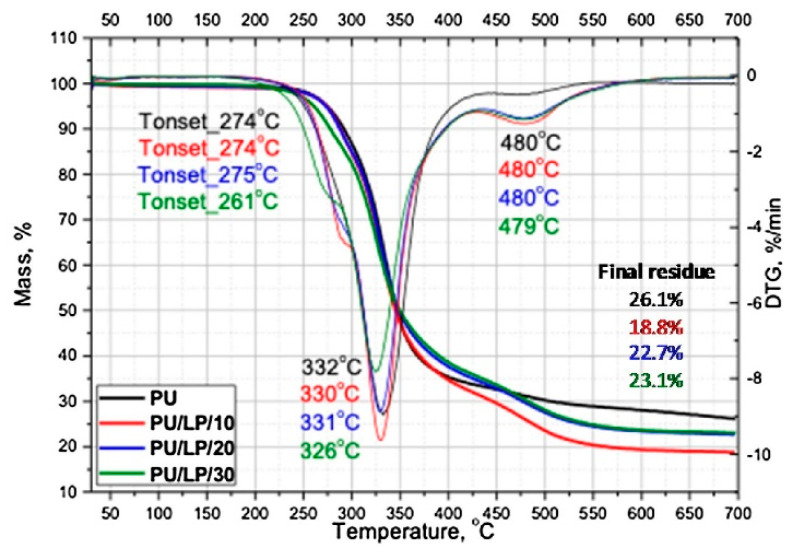
Thermal degradation of PU/LP and pure PU materials [58].

Kurańska et al. [58] made porous PUF composites by mixing a bio-based polyol, synthesized, from lignin with PUF (PU/LP). The results showed that the lignin–PUF composites exhibited a rapid reduction in terms of dielectric polarization during foaming, which led to a slight increase in the reactivity of the whole system, whereas the enhanced thermal stability and degradation behavior made the thermal properties of the composite foam better than that of petrochemical-derived foams. Finally, its thermal insulation coefficient was 0.23 W/(m·K), which showed a better performance in terms of thermal insulation and heat preservation. Thermogravimetric analysis (TGA) was used to investigate the thermal degradation process of the obtained materials. The TGA was carried out using NETZSCH (model TG 209F3, Tarsus, Selb, Germany) equipment. Approximately 8–10 mg of RPU foam was heated in alumina crucibles at temperatures ranging from an ambient temperature to 700 °C, at a constant heating rate of 10 °C·min^−1^. Figure 11 shows the TGA profiles of all the RPU foams in an N_2_ atmosphere. All foams were thermally stable up to 261–276 °C, with PU/LP/30 having the least stability.

As global efforts toward sustainable materials intensify, bio-based rigid PU foams offer a promising solution. The synthesis of bio-based PU foams involves replacing or partially substituting conventional petrochemical-derived polyols with renewable alternatives sourced from agricultural and forestry residues. These alternatives, such as soybean oil, castor oil, and lignin, hold promise for creating environmentally friendly rigid PU foams. Numerous studies [59,60] have demonstrated the feasibility of utilizing bio-based polyols in foam formulations, showcasing their potential to enhance the foam’s properties while reducing the environmental footprint. By optimizing the processing conditions and foam formulations, researchers aim to achieve mechanical, thermal, and fire-resistant properties, similar to those of traditional petrochemical foams.

Verdolotti et al. [61] prepared an organic–inorganic PUF composite using the sol–gel method, which exhibited properties superior to those of pure PUF materials, such as enhanced thermal stability and thermal insulation. It exhibited better properties than PUF alone by combining the organic and inorganic phases, and the composite structure had nanoscale and multiscale pore characteristics. The layered combination of these pore structures also significantly reduces the thermal conductivity, which reduced to 0.025 W/(m·K) due to the “aerogel-like” polysiloxane structure that was incorporated through the synthesis of this hybrid foam. TGA was performed to evaluate the silica content in the hybrid foams and the effect of silica content on the thermal properties of the hybrid foams, using a TGA Q500 (TA Instruments, New Castle, Delaware, USA), over a temperature range from 30 °C to 1000 °C, in an atmosphere comprising air. DSC analyses were performed to obtain insights concerning the glass transition temperature and the effect of the inorganic phase on this temperature. DSC was performed using Q1000 equipment (TA Instruments, USA). The samples were first heated from −80 to 180 °C, then cooled to −80 °C, and finally reheated to 180 °C. The rate of both heating processes was 10 °C/min. As can be seen in Table 3, the maximum decomposition temperature of the hybrid foam, dominated by T_max2_ and T_max3_, increased with the addition of polysiloxane, compared with the pure PUR. This improvement in thermal stability can be attributed to the structural domain of polysiloxane, which protected the PU phase from thermal degradation and thermal oxidation.

**Figure 12:**
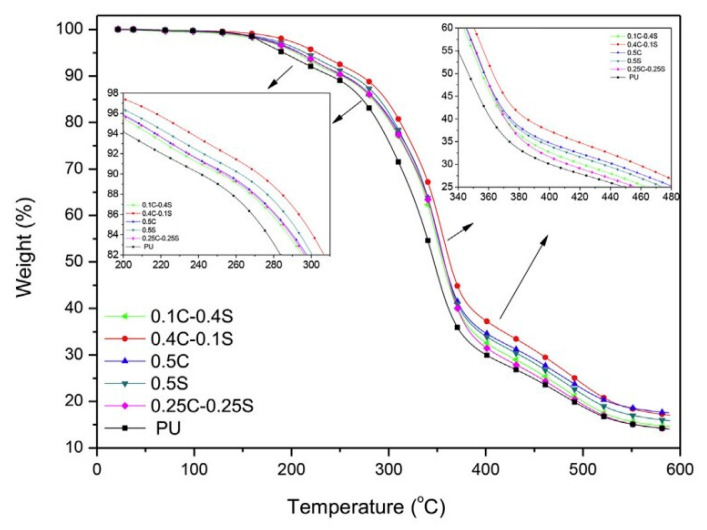
TGA results of pure RPUF and its nanocomposites [62].

Burgaz et al. [62] prepared multiwalled carbon nanotube–RPUF nanocomposites containing hydrophilic-fumed nanosilica with a high surface area and carboxylic acid functionalization using the reactive foaming method and hydrogen bonding interactions between the nanofillers and the polymer matrix. The thermal decomposition of the PU nanocomposites was determined using Simultaneous Differential Thermal Analysis (DSC–TGA) using Q600 equipment (TA Instruments). The samples were heated in alumina pans at temperatures ranging from 20 °C to 600 °C, at a heating rate of 20 °C/min, under a flow of N_2_ gas. An empty alumina pan was used as a reference for each test. The results showed that the addition of silica nanoparticles and carbon nanotubes to RPUFs improved their thermal stability (the TGA results of RPUF composites are shown in Figure 12), and the system containing 0.4 wt% carbon nanotubes and 0.1 wt% silica nanoparticles had higher thermal stability compared with the samples containing 0.5 wt% carbon nanotubes and 0.5 wt% silica nanoparticles.

### 4.4. Characterization of the Flame-Retardant Properties

FPUF has high flammability, a fast combustion rate, and a high exothermic rate, and it generates a large amount of smoke and toxic volatiles such as isocyanates, which can easily lead to disastrous fires and cause casualties. Therefore, improving the flame-retardant performance of FPUF, and analyzing its dangerous products when pyrolysis occurs, have received much attention. The flame-retardant performance of FPUF must meet the requirements of national or international standards and the final applications. The flame-retardant characteristics of a PUF can be divided into two types, as follows: additive flame retardants and reaction flame retardants.

**Figure 13:**
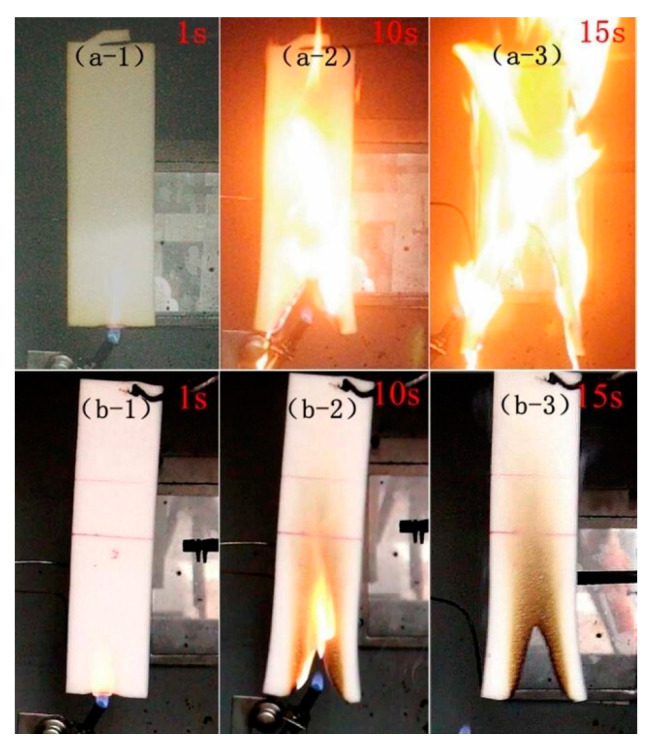
Digital photographs of the original FPUF (**a-1**–**a-3**) and FPUF materials mixed with DPPMA (**b-1**–**b-3**) at different ignition times [65].

**Figure 14:**
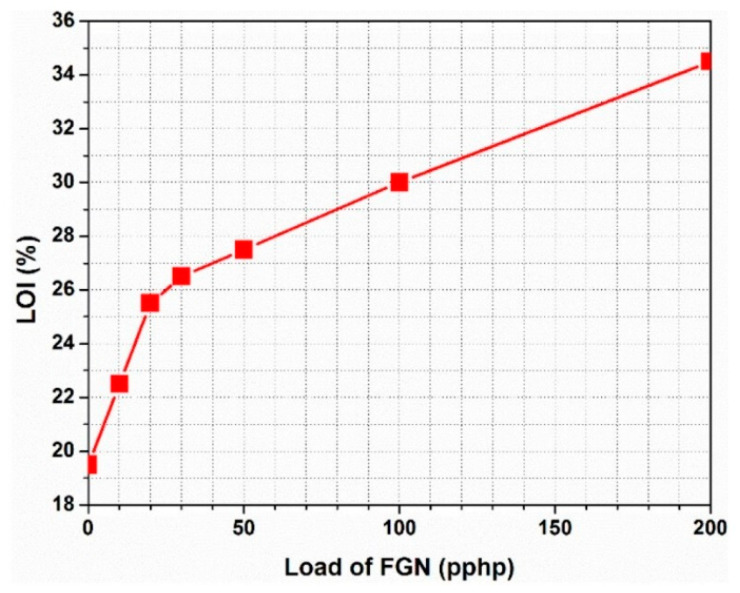
LOI of PUF with different FGN contents [66].

Additive flame retardants, including halogenated flame retardants, phosphorus-based flame retardants, nitrogen-based flame retardants, and inorganic flame retardants, were added to the raw materials of FPUF via physical addition. Some studies have proven that halogen-containing compounds have flame-retardant effects on FPUFs, but the release of black smoke and potential toxic and corrosive gases during the combustion of halogen-containing compounds limits their application [63]. Considering the adverse effects of halogenated flame retardants, an increasing number of halogen-free flame retardants are being used in large quantities. Among them, phosphorus-containing compounds and graphene have been widely investigated for their high flame-retardant efficiency. Akdogan et al. [64] prepared composite RPUF materials containing triphenyl phosphate (TPhP), aluminum trihydrate (ATH), and cyclopentane/isopentane blends as blowing agents using a simple hand-stirring method. The composite had excellent flame-retardant properties, and the ultimate oxygen index (LOI) of the composite RPUF materials also increased from 19.3% to 21.9%, 22.2%, and 21.7% of the original RPUF when 50A, 50T, and 20A/20T were added, respectively. Rao et al. [65] synthesized an efficient flame-retardant melamine salt (DPPMA), composed of diphenyl phosphonic acid (DPPA) and melamine (MA), and applied it to an FPUF. The combustion tests (including LOI, CC, and vertical combustion tests) showed that the flame-retardant properties of the FPUF were significantly improved with the addition of DPPMA. The vertical combustion test (see Figure 13) showed that the FPUF could self-extinguish when the DPPMA content in the FPUF was only 5 wt%. These results indicated that DPPMA has excellent flame-retardant properties for FPUFs. Cao et al. [66] designed a new epoxy resin containing phosphorus [67] and modified functionalized graphene (FGN). Since the epoxy group of functionalized graphene can react with the isocyanate group during the foaming process of PU, the functionalized graphene has good compatibility with the PU matrix. After synthesis, the composite foam material had excellent characteristics, including a high flame-retardant ability and smoke suppression. The LOI value increased rapidly when the FGN loading was below 6.1 wt%, and a further increase in FGN led to an increase in the LOI value, but the rate decreased. When the FGN loading was 11.5 wt%, the LOI was 30%. When the FGN loading was 20 wt%, the value increased further to 34.5% (see Figure 14). Moreover, since functionalized graphene can significantly accelerate the formation of carbonized slag at high temperatures, functionalized graphene may also reduce the total smoke production of the composite foam, and the total amount of smoke emitted by the composite material was reduced from 6.11 m^2^/m^2^ for the pure PU material to 2.08 m^2^/m^2^, which gave the material better smoke suppression and fire resistance overall.

The use of reactive flame retardants offers many advantages over additive flame retardants, including the increased compatibility of the flame retardant with the polymer, which provides permanent flame-retardant abilities and it offers high flame-retardant efficiency [68]. The reactive flame retardants commonly used in PU materials are polyols or isocyanates containing phosphorus, silicon, and nitrogen [69]. Zhang et al. [70] synthesized nitrogen-containing reactive flame retardants using polymethyl cyanamide (HM), and they prepared a flame-retardant PUF using HM and dimethyl phosphate (DMMP). The results showed that the addition of the HM/DMMP flame retardant made the charred layer of PUF denser, thus blocking the transfer of heat and flame, and it achieved a good flame-retardant effect. The LOI value of this PUF could reach up to 31.65% and it passed the UL 94V–0 vertical combustion test. Bhoyate et al. [71] synthesized reactive flame-retardant polyols based on phenyl phosphonic acid and propylene oxide, and they used them together with limonene-based polyols for the preparation of flame-retardant PUFs. The addition of phosphate improved the compressive strength of the foam, and the graphite in the coke layer prevented the combustible medium from contacting oxygen and prevented the transfer of heat, thus improving the flame-retardant effect. Liang et al. [72] investigated the effect of different organophosphorus compounds (phosphate and phosphoramidite) on the flame-retardant properties of PUF, and the results showed that phosphoramidite could enhance the flame-retardant effect of PUF, but more PO and PO_2_ radicals would be released during combustion. Zhang et al. [73] synthesized a new phosphorus-containing nitrogen reactive flame retardant using DOPO, paraformaldehyde, and diisopropanolamine as the raw materials, and the PUF; this flame retardant exhibited significant resistance to heat. The reactive flame retardant overcame the disadvantages of additive flame retardants such as easy migration, the inability to maintain the flame-retardant effect, and the destruction of the physical properties of the foam.

## 5. Conclusions and Prospects

PTIMs have become a popular research topic today because of their excellent thermal insulation properties and wide range of applications. With the development of society, the standards for the performance of thermal insulation materials are also increasing. PUPM is used in a variety of applications, and it is a lightweight, non-toxic polymer material, with very broad prospects for application. Depending on the practical applications, a variety of porous materials with different shapes, pore sizes, pore shapes, and porosities can be prepared using a variety of methods. 

However, each preparation method has certain limitations, such as the difficulty of selecting the particle size for solution casting, the difficulty of selecting porous compounds for melt formation, leaching, high–temperature operations, and the instability of lyophilized emulsions. Therefore, the problem that needs to be solved now concerns the combination of different preparation techniques with complementary advantages in order to improve the preparation process, eliminate the limitations, and meet the practical requirements of applying PUPMs.

For the porous thermal insulation material represented by PUF, which has excellent characteristics, but high flammability and it releases toxic gas during combustion, we need to choose a suitable polymer after synthesizing the PU so that the final composite material still has the good thermal insulation properties of the original PU (or better), and at the same time, it can ensure excellent mechanical properties and flame-retardant properties to meet the diverse needs of porous thermal insulation materials in various fields in the future. There will be a bright future in terms of the application of porous thermal insulation materials, as they will comprise excellent heat insulation, mechanical, and flame-retardant properties.

## Figures and Tables

**Table 1:** Thermal conductivity of the RPUF nanocomposites, W/(m·K) [50].

Silica Aerogel (wt%)	Blowing Agent			
	CFC–11	Cyclopentane	Normal Pentane	Normal Hexane
0%	0.0209 ± 0.0003	0.0314 ± 0.0001	0.0301 ± 0.0001	0.0345 ± 0.0002
1%	0.0189 ± 0.0002	0.0295 ± 0.0003	0.2879 ± 0.0002	0.0328 ± 0.0001
3%	0.0178 + 0.0001	0.0277 ± 0.0002	0.0266 ± 0.0001	0.0308 ± 0.0002
5%	0.0171 ± 0.0002	0.0268 ± 0.0001	0.0257 ± 0.0001	0.0299 ± 0.0001

**Table 2:** Mechanical properties and thermal conductivity of the pure PUF and SiO_2_/PUF composites [51].

Sample	Density (kg/m^3^)	Weight Gain (wt%)	Compressive Strength (kPa)	Thermal Conductivity mW/(m·K)
Pure PUF	28.5 ± 0.1	/	221 ± 13	30.9
SiO_2_/PUF–1	32.2 ± 0.8	13.0	335 ± 26	29.6
SiO_2_/PUF–3	34.7 ± 0.6	21.8	418 ± 22	29.0
SiO_2_/PUF–5	37.9 ± 1.2	33.0	486 ± 37	28.2

**Table 3:** Thermal stability of the PUF material [61].

Sample	T_max1_	T_max2_	T_max3_	Residue at 1000 °C, wt% SiO_2_	Tg (°C)	λ (W/mK)
Pristine PUR	316	330	560	0	24.5	0.04
HPUR_wca_	273	314	561	17	40	0.032
HPUR_ca1_	215	335	570	17.8	49.1	0.028
HPUR_ca2_	267	340	620	20	59.2	0.025

## Data Availability

Data are available on request due to restrictions (e.g., privacy or ethical).
